# Genetic origin of goat populations in Oman revealed by mitochondrial DNA analysis

**DOI:** 10.1371/journal.pone.0190235

**Published:** 2017-12-27

**Authors:** Nasser Ali Al-Araimi, Osman Mahgoub Gaafar, Vânia Costa, Agusto Luzuriaga Neira, Raed Mahmoud Al-Atiyat, Albano Beja-Pereira

**Affiliations:** 1 Research Center in Biodiversity and Genetic Resources (CIBIO), University of Porto, Vairão, Porto, Portugal; 2 Department of Animal & Veterinary Sciences, College of Agricultural & Marine Sciences, Sultan Qaboos University, Al-Khod, Sultanate of Oman; 3 Department of Animal Production, Faculty of Agriculture, Mutah University, Al-Karak, Jordan; Universita degli Studi di Pavia, ITALY

## Abstract

The Sultanate of Oman has a complex mosaic of livestock species and production systems, but the genetic diversity, demographic history or origins of these Omani animals has not been expensively studied. Goats might constitute one of the most abundant and important domestic livestock species since the Neolithic transition. Here, we examined the genetic diversity, origin, population structure and demographic history of Omani goats. Specifically, we analyzed a 525-bp fragment of the first hypervariable region of the mitochondrial DNA (mtDNA) control region from 69 Omani individuals and compared this fragment with 17 mtDNA sequences from Somalia and Yemen as well as 18 wild goat species and 1,198 previously published goat sequences from neighboring countries. The studied goat breeds show substantial diversity. The haplotype and nucleotide diversities of Omani goats were found equal to 0.983 ± 0.006 and 0.0284 ± 0.014, respectively. The phylogenetic analyses allowed us to classify Omani goats into three mtDNA haplogroups (A, B and G): haplogroup A was found to be predominant and widely distributed and accounted for 80% of all samples, and haplogroups B and G exhibited low frequencies. Phylogenetic comparisons with wild goats revealed that five of the native Omani goat populations originate from *Capra aegagrus*. Furthermore, most comparisons of pairwise population *F*_*ST*_ values within and between these five Omani goat breeds as well as between Omani goats and nine populations from nearby countries were not significant. These results suggest strong gene flow among goat populations caused by the extensive transport of goats and the frequent movements of human populations in ancient Arabia. The findings improve our understanding of the migration routes of modern goats from their region of domestication into southeastern Arabia and thereby shed light on human migratory and commercial networks during historical times.

## Introduction

Domesticated goats (*Capra hircus*) are among the most important farm animals in many parts of the world, particularly Africa and Asia. Goats were thought to have been domesticated in the ninth and eighth millennia BC during the Pre-Pottery Neolithic B (PPNB) period, specifically between 8700 and 6800 cal BC in the Zagros Mountains, which forms part of modern Iran [1], as well as in the high Euphrates valley and southeastern Anatolia [2]. Known for their hardiness, goats have adapted to many different habitats, including savannas, deserts, scrubs and mountain ranges, and are found in all types of ecological environments, particularly in tropical and dry regions, where other livestock species have difficulties surviving [3]. Goats are included in the entire range of production systems, from intensive production to smallholders and nomadic human populations [4], and are raised around the world for their meat, milk, cheese, fiber and hides. Goats are also kept as pets or for entertainment or cultural and religious purposes.

In early history, the present-day Sultanate of Oman was located in a very strategically valuable region of southeastern Arabia. In fact, the northern coast of Oman was a “commercial hub” among the Fertile Crescent, the Indus Valley and the northeast coast of Africa. The southern part of Oman was an important region for frankincense, and it has been hypothesized that the dromedary camel underwent domestication somewhere in the southern region between Oman and Yemen [5]. The complex mosaic environment of the southeastern Arabian Peninsula is key to understanding its history of occupation by humans and animals. In Oman, goats are the most abundant livestock species and represent an integral part of the farming system and agricultural economy. The estimated population of goats is approximately 2.1 million (64%) among a total of 3.2 million heads of livestock [6], and goats in Oman include four recognized breeds, namely, Jabal Akhdar, Batinah, Dhofar and Ash Sharqiyah, and additional nondescript populations. In addition, several local goat strains (including Al Saidi, Al Rahbi, Al Jamodi, Ramli, Jabali and Musandam goats) are well adapted to the different agro-climatic conditions of various regions. However, the productive and reproductive performances, growth rate, meat quality, and milk production of these strains are not well known, and no genetic evaluation, identification or improvement programs have been implemented for these populations.

The history of native goats in Oman is long and complex and remains a subject of dispute. Nonetheless, evidence of the ancient presence of goats based on ancient rock engravings and paintings of domesticated goats, likely from the fifth and fourth millennia BC (7,000–6,000 years ago), can be found in many caves and rocks throughout Oman [7,8]. According to archaeological research, goats were already present in the faunal assemblages of several sites in Oman toward the end of the seventh millennium BC [9,10]. Regardless, the history of goats in Oman and throughout the Arabian Peninsula is complex, and very little information is currently available. Specifically, there is no evidence of the goat’s wild ancestor (*C*. *aegagrus*) [9], and the routes through which the domestic goat was introduced into Oman have been poorly studied.

Mitochondrial genome (mtDNA) analysis is a critical tool in the fields of evolutionary and population genetics, including molecular ecology. Indeed, the mtDNA diversity has been proven to be particularly useful in assessing the origin, phylogeny, maternal lineage and population structure of many livestock species throughout the world [11–14]. Previous studies of goat mtDNA diversity have revealed six highly divergent mtDNA haplogroups in domestic goats: A, B, C [14], D [15], F [16] and G [17]. Haplogroup A is the most frequent, and a study of a large series of samples collected from a wide geographic distribution revealed that this haplotype is present in more than 90% of goats. In contrast, the population sizes of the other haplogroups are substantially smaller (< 7%) and have limited distribution areas [17,18]. Recent molecular studies found that the major haplogroups (A, B, C and D) underwent between the Middle Paleolithic and Epipaleolithic periods (91,000–9,000 years ago), significantly predating the beginning of domestication (10,000 years ago) [18,19].

Three wild species of the genus *Capra*, the bezoar (*C*. *aegagrus*), markhor (*C*. *falconeri*) and ibex (*C*. *ibex*), are closely related to the domestic goat (*C*. *hircus*), and it has been suggested that these species contributed to the gene pool of domestic goats [20–23], possibly via early translocations of animals and/or feralization prior to the global spread of goats [17,23]. It is now widely agreed that *C*. *aegagrus* is the wild ancestor of domestic goats, and this finding has been confirmed by the presence of all “domestic” haplogroups in the current *C*. *aegagrus* populations [18,23].

Due to the lack of an extensive evaluation of the genetic diversity within Omani goat breeds, we analyzed four economically important Omani goat breeds and one additional strain. All of the studied strains are phenotypically distinct from each other and have very different geographic ranges. The genetic diversity and distribution of mitochondrial haplogroups among these breeds were examined, and the level of maternal introgression of goat populations from different putative domestication centers was also assessed. Finally, to support our genetic data and initiate a detailed documentation of *Capra* biodiversity in Oman, the hypothesis that *C*. *aegagrus* is the wild progenitor of the Omani domestic goat was evaluated.

## Materials and methods

### Ethics statement

Animals from five Omani provinces were sampled. With the consent of the flock owners (villagers), tissue samples were collected from native goats by veterinarians or under veterinary supervision during routine veterinary care. We obtained permission to use the facilities of the Director General of Animal Wealth of the Ministry of Agriculture and Fisheries Wealth in Oman.

### Sampling

**Fig 1:**
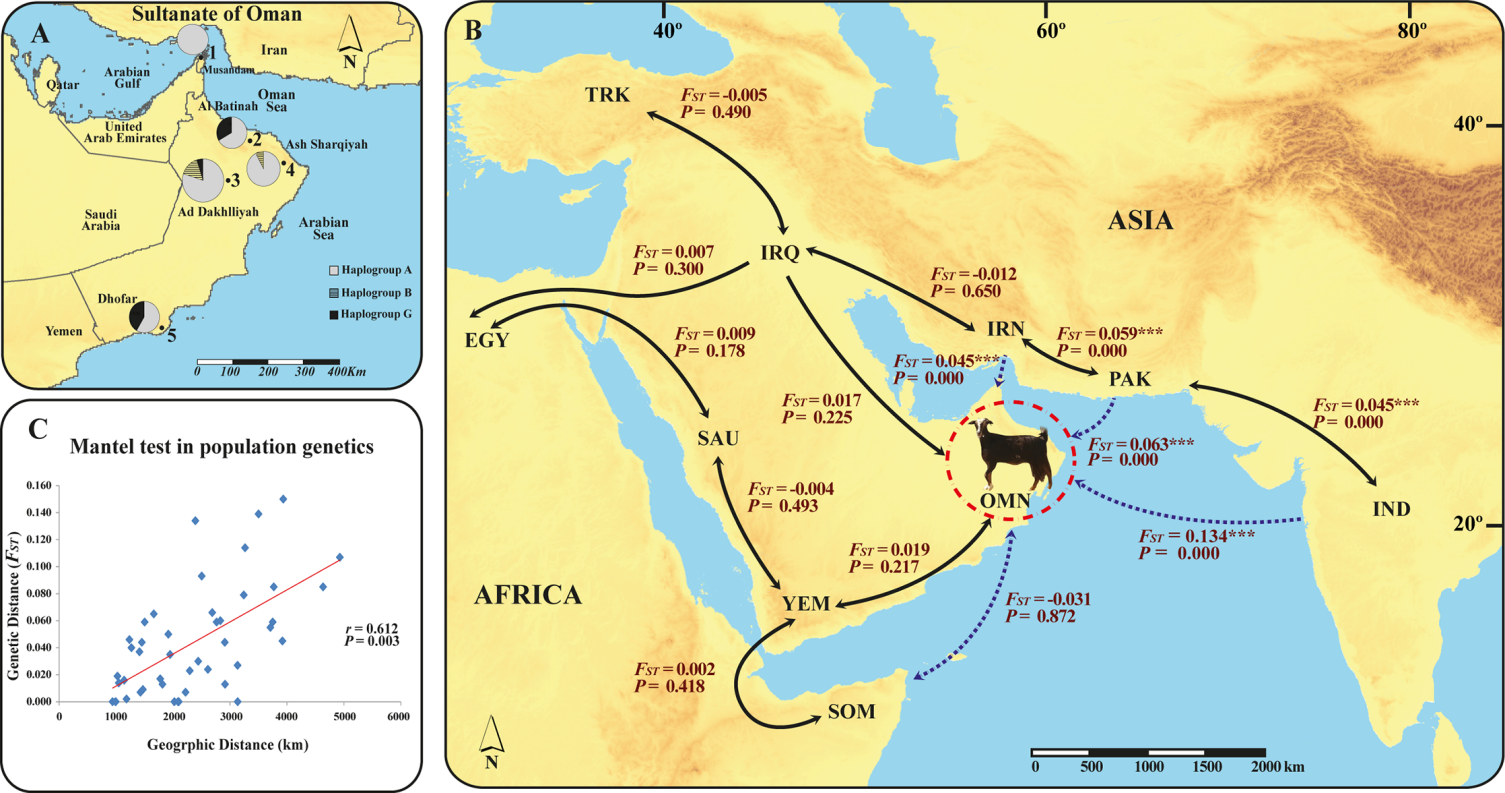
**(A)** Sampling sites and approximate geographic distributions of the three mitochondrial (mtDNA) haplogroups of Omani goats. The sampling collection sites are indicated on the map with darker dots. The figures are marked on the map according to the names of the collection sites: 1, Dibba; 2, Al Khaburah; 3, Al Jabal Akhdar; 4, Jalan Bani Bu Ali; and 5, Marbat. The pie charts show the relative proportions of the A, B and G haplotypes observed in samples from across the Oman region. The circle sizes are proportional to the number of goats, and each specific haplogroup is represented by a different color. **(B)** Pairwise *F*_*ST*_ values measured between Omani goats and neighboring goat populations. The abbreviations correspond to the analyzed populations and are provided in S2 Table. The arrows indicate the proposed route for the introduction of goats into Oman from putative domestication areas. Solid black lines represent continental routes, and dashed blue lines represent maritime routes. The statistical significance is indicated as follows: * *P*-value<0.05; ** *P*-value<0.01; *** *P*-value<0.001. **(C)** Scatterplot showing the relationship between pairwise *F*_*ST*_ values and geographic distances (r = 0.612; *P* = 0.003) for the ten domestic goat populations.

Sampling was conducted in four different geographical regions of Oman (A’Dakhiliyah, Al Batinah, Ash Sharqiyah and Dhofar), which are the locations where breeding of the main native Omani goat breeds, Jabal Akhdar (n = 20), Batinah (n = 12), Dhofar (n = 12) and Ash Sharqiyah (n = 13), is concentrated. The Musandam goat strain (n = 12) from northern Oman (Musandam peninsula) was also examined. Brief descriptions of the Omani goat populations are provided in S1 File. S1 Fig presents the morphological characteristics of the five populations, and the geographic locations of all sampling sites for the five populations are shown in Fig 1A. Ear tissue biopsies were obtained by clipping the perimeter of the ear pinna where the tissue is thinnest using an ear notcher. Each tissue biopsy was stored at -20°C in 95% ethanol prior to DNA extraction. To avoid any close genetic relationships (parents and grandparents), only two unrelated animals from each herd were sampled. Care was taken to distinguish pure native breeds from crossed goats or commercial breeds (e.g., cosmopolitan breeds) as well as from nondescript breeds using knowledge and information provided by goatherds and villagers.

### DNA extraction, PCR amplification and sequencing

Genomic DNA from skin tissue was extracted using DNeasy Blood and Tissue Kit (Qiagen, Hilden, Germany) according to the manufacturer’s recommended protocols. We amplified a 598-bp fragment of the mitochondrial hypervariable region 1 (HV1) between positions 15,653 and 16,250 in the reference goat mitochondrial genome AF533441 [24] using the following two internal primers: CAP-F (5′-CGTGTATGCAAGTACATTAC-3′) and CAP-R (5′-CTGATTAGTCATTAGTCCATC-3′). Polymerase chain reaction (PCR) amplification was performed in a final volume of 20 μL, which included 0.8 μM of each primer, 2.5 mM MgCl_2_ (50 mM), 0.3 mM dNTPs (10 mM each), 0.25 U of Platinum Taq Polymerase (Applied Biosystems, USA), 1X PCR buffer, 0.3 μg/μL bovine serum albumin (BSA; 50 μg/μL) and variable amounts of genomic DNA. All reactions were performed with negative controls. The PCR conditions for amplification consisted of a pre-denaturation step of 94°C for 10 min followed by 40 cycles of 94°C for 30 s, 61°C for 30 s and 72°C for 30 s and a final extension step at 72°C for 5 min. The PCR products were examined on 1% agarose gels and sequenced with an ABI 3130xL capillary sequencer using a Dye Terminator Cycle Sequencing Reaction Kit (Applied Biosystems, USA).

Both forward and reverse DNA sequences of the 69 Omani goats were examined, edited, aligned and trimmed manually to produce a single concatenated file with a consensus region of approximately 598-bp. The concatenated sequences were aligned against the reference sequence AF533441 [24] and further trimmed using ClustalW implemented in the MEGA6 program [25]. The final dataset comprises a 525-bp fragment corresponding to the region between positions 15,687 and 16,211 of the *C*. *hircus* mtDNA reference sequence. One fragment from the Jabal Akhdar goat was 480-bp, and the 45 missing nucleotides were treated as missing data. The sequences were deposited at GenBank under the accession numbers KY399058-KY399142 and KY449044.

To provide more complete information regarding the origin of Omani goats, new mtDNA sequences from Somalia (n = 8; collected from Hargeysa) and Yemen (n = 9; collected from Aden) were included, and mtDNA sequences from wild and domesticated goats were also downloaded from GenBank (S1 and S2 Tables). Because many published mtDNA goat sequences are substantially shorter or longer than our sequence (525-bp), the aligned sequence was assembled into three datasets. The set used for the first analysis included Omani goat samples and 22 goat reference sequences (454-bp; region between positions 15,735 and 16,188) belonging to the six known haplogroups (A, B, C, D, F and G) [17]. These sequences were included to facilitate recognition of the haplogroup frequency status of each individual goat and within each breed. The second set of sequences (480-bp; region between positions 15,707 and 16,186) was used to elucidate relationships with 18 sequences from seven wild goat species (S1 Table). The final set of sequences was used for comparisons between Omani goats and nine other populations (Somalia, n = 8; Yemen, n = 9; Iraq, n = 7; Saudi Arabia, n = 44; Egypt, n = 29; Iran, n = 219; Turkey, n = 349; Pakistan, n = 88; and India, n = 462) from possible domestication centers and neighboring countries (440-bp; region between positions 15,747 and 16,186). All fragments of the three datasets were aligned and trimmed using ClustalW in MEGA6 [25].

### Statistical analysis

For each goat breed, Arlequin ver. 3.5.1.2 [26] was used to compute the following parameters of mitochondrial polymorphisms: number of haplotypes (Hn), number of polymorphic sites (S), haplotype diversity (Hd), nucleotide diversity (π) and its standard deviation (SD), and mean number of pairwise differences (*k*). The differentiation within Omani goat breeds was assessed based on pairwise differences (*F*_*ST*_) and through analysis of molecular variance (AMOVA) using Arlequin ver. 3.5.1.2 [26] with 1,000 replications.

To identify possible haplogroups of each individual Omani goat according to reference sequences [17], phylogenetic trees were constructed with the neighbor-joining (NJ) method [27] based on the Kimura 2-parameter model [28] using MEGA6 [25]. Metrics of phylogenetic tree reliability were assessed using 2,000 bootstrap replicates [29], with an alpha value of 0.29 for the goat mtDNA gamma shape parameter [14,17,30]. All positions containing gaps and missing data were eliminated. This dataset was also used to create a phylogenetic tree (NJ) in MEGA6 based on Omani goat sequences and 18 published sequences (in GenBank) of wild goats belonging to seven species (S1 Table). Finally, because networks allow visualization of the relationships between haplotypes and can help reveal how haplotypes are related to each other [31], the population structure of Omani goats was characterized through network analysis. A haplotype median-joining network was generated using PopART ver. 1.7 (http://popart.otago.ac.nz/index.shtml) with the default settings.

Additionally, population pairwise *F*_*ST*_ values among the Omani goat populations and nine other populations were calculated using Arlequin ver. 3.5.1.2 [26]. The statistical significance of the correlations between the pairwise genetic distance (*F*_*ST*_) and the geographic distance (km) based on mtDNA data from the ten populations was evaluated through the Mantel test implemented in GenAlEx ver. 6.501 [32] using 9,999 permutations. To determine the distance between two points (cities) on a map, the geographic distances between populations were calculated using FreeMapTools (https://www.freemaptools.com/how-far-is-it-between.htm). S3 Table shows the relative geographic distances between selected countries. For the analyses, all negative *F*_*ST*_ values were set to zero.

Signs of historical demographic expansions were also explored with Arlequin ver. 3.5.1.2 [26] using two different approaches. First, to establish the presence of population expansion, we used two neutrality tests, Tajima’s D [33] and Fu’s *Fs* [34], to assess significance with 1,000 permutations. Second, historical demographic expansion was investigated based on probability values for the null model of sudden expansion, with parametric bootstrap values from 1,000 iterations. This test included five parameters: the sum of squared deviation (SSD), the raggedness index (Rg) [35], θ_0_ and θ_1_ (before and after population growth), and τ (time since expansion, expressed in units of mutational time). In addition, mismatch frequency graphs were generated to evaluate possible historical events of population expansion or a stationary population history [33]. Finally, the times in years since the expansion (*t*) of Omani goats and each haplogroup were roughly estimated using parameters of demographic expansion with the equation *t* = τ/2*u*. In this equation, τ is the empirical peak of the mismatch distribution, and *u* is the mutation rate of 6.09_X_10^−4^, which was estimated based on the dataset published by Colli et al. [18] with the equation *u* = *m*_*t*_μ, where *m*_*t*_ is the length of the sequence and μ is the substitution rate, μ = 3.95_X_10^−8^. The τ values and their confidence intervals were obtained with Arlequin ver. 3.5.1.2 using 1,000 bootstrap replications [26].

## Results

### Sequence variation

We examined hypervariable region 1 (HV1) fragments of the mitochondrial DNA control region in 69 goats belonging to five different goat populations and representing five different geographic regions of Oman. An analysis of the combined dataset of sequences of Omani goats revealed high polymorphism, with the presence of 45 different maternal haplotypes and 94 polymorphic sites in the 525-bp aligned fragment. The polymorphic sites include 87 transitions, two transversions and five indels, with a ratio of transition to transversion of 43.5. The average base composition is as follows: A, 30.19%; T, 30.97%; G, 16.18%; and C, 22.66%. Thus, the percentage of A+T nucleotide pairs is higher than that of C+G pairs in this region (61.16% and 38.84%, respectively). Haplotype H38 was found to exhibit the highest frequency; specifically, this haplotype was observed in five different individuals from two distinct populations: Musandam and Ash Sharqiyah.

### Genetic diversity

Genetic diversity indices based on the mtDNA HV1 region sequences showed substantial variation among Omani breeds (Table 1). An analysis of haplotype diversity and nucleotide diversity revealed high diversity in the Omani goat populations (Hd = 0.983; π = 0.0284). Among Omani breeds, the number of haplotypes found in each breed ranged from six to 16. The Jabal Akhdar goat (JKH) breed showed 16 haplotypes, which is a higher number than those found in any other breed. Higher numbers of polymorphic sites were found for Ash Sharqiyah (SHR) and JKH (64 and 63, respectively) than for the other Omani goat breeds, and higher haplotype diversity values (Hd = 0.974) were found for these two breeds than for the other populations. Dhofar (DHR) and JKH goats exhibited higher nucleotide diversity (π = 0.0309 and π = 0.0308, respectively), whereas the Musandam goat (MSN) strain was found to be the least variable population (Hd = 0.849; π = 0.0159). The mean number of pairwise differences (*k*) for DHR and JKH goats was higher than those for all other breeds (*k* > 16), and the number of haplotypes (Hn), number of polymorphic sites (S) and mean number of pairwise differences (*k*) for the MSN strain (at 6, 19 and 8.333, respectively) were lower than those for the other Omani goat breeds.

**Table 1:** Genetic diversity indices and distribution of mtDNA haplogroups for Omani goat populations.

Population	Abbreviation	N^1^	Hn^2^	S^3^	Hd^4^ ± SD^5^	π^6^ ± SD	*k* ^7^	Haplogroups^8^
**Jabal Akhdar**	JKH	20	16	63	0.974 ± 0.025	0.0308 ± 0.016	16.142	A(16), B(3), G(1)
**Batinah**	BTN	12	8	39	0.894 ± 0.078	0.0270 ± 0.015	14.136	A(8), G(4)
**Dhofar**	DHR	12	10	50	0.955 ± 0.057	0.0309 ± 0.017	16.212	A(7), G(5)
**Ash Sharqiyah**	SHR	13	11	64	0.974 ± 0.039	0.0276 ± 0.015	14.487	A(12), B(1)
**Musandam**	MSN	12	6	19	0.849 ± 0.074	0.0159 ± 0.009	8.333	A(12)
**Omani Goats^**9**^**	OMN	69	45	94	0.983 ± 0.006	0.0284 ± 0.014	14.883	A(55), B(4), G(10)

^1^Number of individuals.

^2^Number of haplotypes.

^3^Number of polymorphic sites.

^4^Haplotype diversity.

^5^Standard deviation.

^6^Nucleotide diversity.

^7^Mean number of pairwise differences.

^8^Haplogroups observed from the neighbor-joining (NJ) analyses (Fig 2A), with the number of haplotypes per haplogroup given in parentheses.

^9^Omani goats are abbreviated as (OMN) and include five populations.

### Phylogenetic analysis

**Fig 2:**
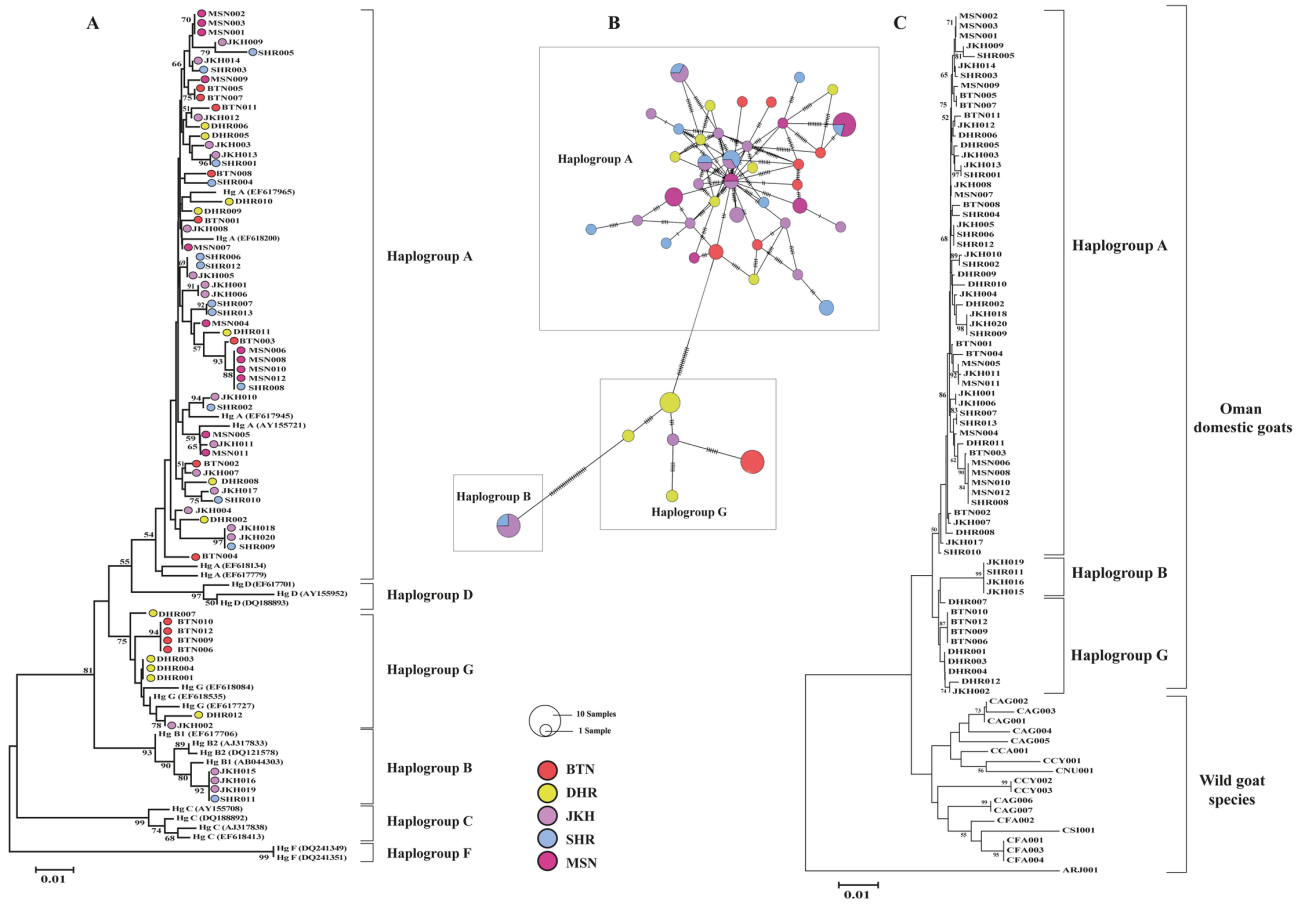
**(A)** A neighbor-joining (NJ) tree of 69 Omani goat sequences and 22 goat reference sequences. The values on the branches represent the bootstrap support based on 2,000 replicas; values lower than 50% are not shown. The GenBank accession numbers for the haplotype references are shown in parentheses. The bar scale indicates the mtDNA genetic distance. **(B)** Median-joining network representing the genetic relationships among mitochondrial haplotypes in the goat populations from Oman. The circles representing the haplotypes are colored according to the sample population and scaled according to the number of supporting sequences. The dashed lines indicate the numbers of mutational steps between two haplotypes. **(C)** NJ tree for five Omani goat populations and 18 wild goat sequences. The numbers at the branches represent the bootstrap values based on 2,000 replicas. Bootstrap values equal to or greater than 50% were considered, and the scale bar for the branch lengths is shown. The breed names are abbreviated as defined in Fig 2A, and the full names of the wild goat species are given in S1 Table. The sample population names are abbreviated as follows: Batinah (BTN), Dhofar (DHR), Jabal Akhdar (JKH), Ash Sharqiyah (SHR) and Musandam (MSN).

The phylogenetic relationships among individuals were investigated using the NJ tree, and the individuals were classified into haplogroups according to a previous classification system [17]. The NJ tree divided all Omani goat sequences into three different haplogroups: A, B, and G (Fig 2A).

Haplogroup A was highly dominant among Omani goats. In fact, 55 of the animals were classified as haplogroup A, which the most widely represented and distributed haplogroup throughout the studied geographic regions. A few samples of Omani goats were clustered into haplogroups B and G. The frequencies of haplogroups A, B and G in the Omani goat population were calculated as 0.80, 0.06 and 0.14, respectively (Fig 1A). Haplogroup B was restricted to the JKH and SHR populations, and the G haplogroup mostly consisted of BTN and DHR goats and one individual from the JKH population and did not include any individuals in the SHR and MSN populations. The JKH population was found to harbor three haplogroups (haplogroups A, B and G), whereas MSN goats in northern Oman show only haplogroup A. The other breeds, which are mostly found in coastal areas, farming and village areas, exhibit two haplogroups (A:B and A:G), as shown in Table 1. The phylogenetic tree revealed similar branching patterns for the breeds, and the data indicate the absence of breed-specific localization or distribution. Additionally, the topology of the median-joining network of Omani goats showed that the haplotypes did not cluster according to population or geographic region; instead, most of the haplotypes were intermingled, as illustrated in Fig 2B. To obtain more complete information regarding the wild progenitor species of Omani goats, we also constructed an NJ phylogenetic tree. According to our genetic data, the wild species with the shortest genetic distance (i.e., branch length) to Omani goat populations was identified as the wild goat *C*. *aegagrus* (Fig 2C).

### Population genetic structure

The genetic differentiation among Omani goat breeds was assessed through pairwise *F*_*ST*_ comparisons (S4 Table), which revealed that the genetic differentiation between Omani breeds is generally moderate (*F*_*ST*_ = 0.005–0.163). The pairwise *F*_*ST*_ comparisons revealed significantly greater (*P*<0.05) genetic differentiation between the MSN goat strain and the other breeds, with the exception of the SHR goat population (S4 Table). Only one of the ten possible comparisons of *F*_*ST*_ estimates, specifically that between JKH and SHR goats, was negative and not significant (*F*_*ST*_ = -0.027; *P*>0.05). A hierarchical AMOVA revealed very low variation among the studied populations within the geographical area of Oman. The AMOVA results indicated that the within-population variation contributes 93.98% of the total variation, whereas the among-population variation contributes only 6.02%of the total variation (*P* < 0.05, S5 Table).

Pairwise *F*_*ST*_ comparisons were then performed between Omani goats and those from neighboring countries. Because these neighboring countries provide the most geographically proximate goat populations, these populations are assumed to have the highest potential for having experienced gene exchange with Omani goats through historical human movement and trade activities, which would account for the gene pool pattern observed. All pairwise population *F*_*ST*_ comparisons showed that the Omani populations are significantly (*P*<0.05) different from all other populations, with the exception of some adjacent or nearby populations (*P*>0.05; S6 Table and Fig 1B). With one exception, all pairwise *F*_*ST*_ values for Omani goats with other populations were positive, ranging from 0.015 (Saudi Arabia, approximately 1,144 km away) to 0.134 (India, approximately 2,394 km away). Additionally, a significant correlation between *F*_*ST*_ values and geographic distances was detected, as indicated by the Mantel test results (r = 0.612; *P* = 0.003; Fig 1C).

### Population demographic history

The mismatch distribution and Fu’s *Fs* statistic, which are the two tools used to infer the demographic history of Omani goats in this study, were applied to detect past population growth (Table 2). A highly negative statistically significant Fu’s *F*_*S*_ statistic value (-14.357) (*P*<0.05) and a low negative Tajima’s *D* value (-0.763) that was not statistically significant (*P*>0.05) were calculated for Omani goats (Table 2).

**Table 2:** Neutrality tests and demographic expansion parameters for Omani goats and their haplogroups.

Population/Haplogroup	Neutrality Tests				Demographic Expansion						
	*D*^1^	*P*^2^	*F*_*S*_^3^	*P*	SSD^4^	*P*	Rg^5^	*P*	τ^6^	θ_0_^7^	θ_1_^8^
**Omani Goats^**9**^**	-0.763	0.234	-14.357	0.002	0.003	0.744	0.005	0.218	5.016	12.243	343.381
**Haplogroup A**	-1.288	0.075	-20.187	0.000	0.002	0.626	0.007	0.383	6.562	3.799	124.219
**Haplogroup G**	0.118	0.606	1.967	0.818	0.067	0.130	0.126	0.178	7.736	0.002	17.842

^1^Tajima's *D*.

^2^Significance values.

^3^Fu's *F*_*S*_.

^4^Sum of squared deviations between the observed and expected mismatch values.

^5^Harpending’s raggedness index of the observed distribution.

^6^Time since expansion in mutational units.

^7^Before population growth (initial/present effective population size).

^8^After population growth (final/past effective population size).

^9^Omani goats include five populations.

**Fig 3:**
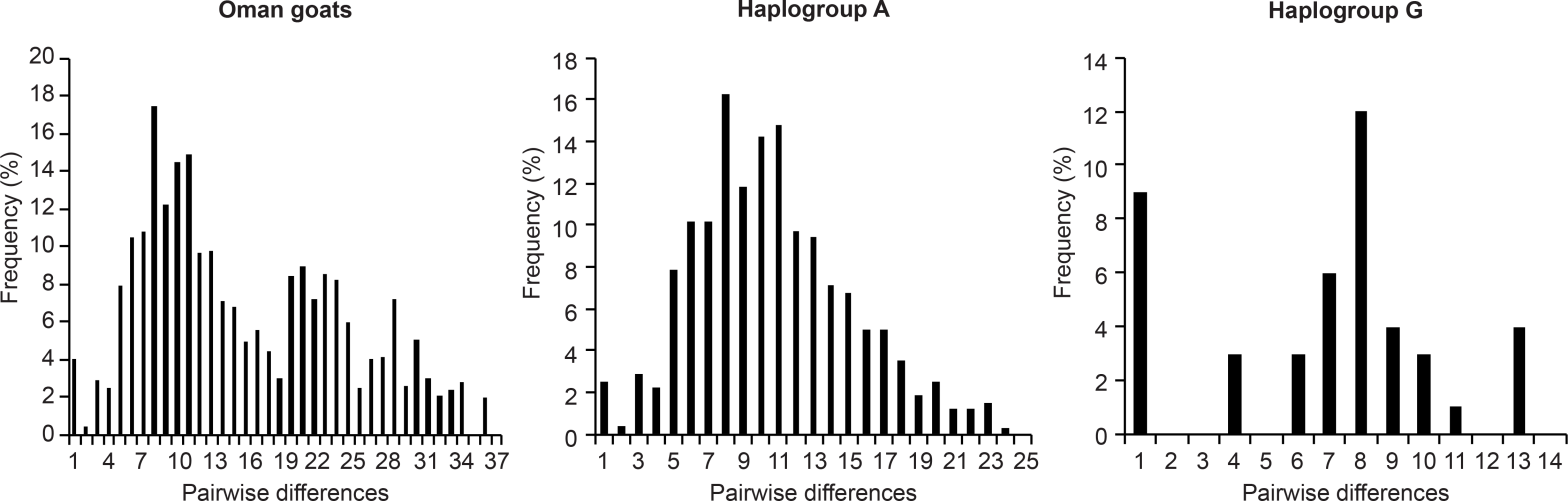
Mismatch distribution graphs for Omani goats and their mtDNA haplogroups.

In addition, the SSD value was 0.003, and Harpending’s raggedness index (Rg) was 0.005. Both of these values were non-significant (*P*>0.05), indicating that the data showed a relatively good fit to the model of population expansion [35]. According to estimates of θ_0_ and θ_1_, Omani goats expanded from a small to a very large population, with a 95% confidence interval (CI) (Table 2). The mismatch distribution chart for Omani goats generated in this study generally showed multimodal mismatch distribution patterns containing two major peaks (with maximum values) at 10 and 21 pairwise differences and one smaller peak at 30 pairwise differences (Fig 3). The results suggest that at least two expansion events occurred during the population demographic history of Omani goats. These peaks were similar to the peaks identified previously for domestic goats throughout the Old World [17,36,37].

To explore the demographic history of the Omani goat population in further detail, we estimated the Fu’s *Fs* values and mismatch distribution for haplogroups A and G and observed a unimodal bell-shaped distribution (Fig 3), similar to previously reported results [17]; haplogroup B was not considered due to its low prevalence. The results support population expansion for haplogroup A (*F*_*S*_ = -20.187), with high significance (*P* = 0.000), but not for haplogroup G (*F*_*S*_ = 1.967, *P*>0.05, Table 2). Tajima’s *D* values were statistically non-significant (*P*>0.05) for each haplogroup, negative (-1.288) for haplogroup A and positive (0.118) for haplogroup G. Additionally, we observed low non-significant Rg and SSD values for both haplogroups (*P*>0.05, Table 2). The estimated initial and past expansion scaled effective population sizes (θ_0_ and θ_1_) for haplogroups A and G were very small, indicating that the mtDNA variation within these two haplogroups originated from a very small doe population.

Finally, we estimated the time since the expansion of Omani goats as well as the minimum and maximum values for expansion times based on 95% confidence intervals. These expansion times, which are expressed in mutational units (τ), were estimated to be 5.016 (CI_95%_: 2.459–23.525) for the Omani goat populations. The approximate time since expansion is therefore 4,119 (2,019–19,319) years. With the exception of haplogroup B due to its single haplotype, we also obtained the time since expansion for each haplogroup in our datasets. The estimated expansion times for haplogroups A (τ = 6.562; CI_95%_: 3.732–16.018) and G (τ = 7.736; CI_95%_: 2.387–12.025) based on 95% confidence intervals are approximately 5,389 (3,065–13,154) and 6,353 (1,960–9,875) years ago, respectively.

## Discussion

The current study constitutes the first assessment of the genetic diversity and population structure of goat populations from the southeastern Arabian Peninsula and provides the first insights into the genetic history of these goat populations. All Omani goat breeds included in our study were observed to show high diversity, as demonstrated by the high number of distinct haplotypes (45) observed in a sample of 69 animals. The genetic diversity parameters of Omani breeds were within the range of those observed for other goat populations from Africa [36] and Asia [37] and were similar to those found for other goat populations in the Arabian Peninsula, specifically Saudi Arabia [17]. A higher genetic diversity would be expected for populations around the Fertile Crescent due to the importance of this region as a domestication center [17], and similar trends have been observed in other species, such as cattle [38], sheep [39] and domestic donkeys [40].

A sequence analysis of Omani goat mtDNA revealed a comparative lack of genetic structure, supporting a geographic distribution that confirms the main trends observed in previous studies of mtDNA diversity in goats [14,17]. AMOVA results showed that 93.98% of the variation occurs within Omani goat populations, whereas only 6.02% occurs between populations. Compared with the AMOVA results obtained for other within-populations datasets [14,17,41], our results also support the hypothesis that modern domestic goats expanded from relatively few haplogroups, with little geographic variation among most extant goat populations [41]. This observation was further supported by a median-joining network, in which the haplotype distribution pattern did not cluster according to population or geographic region (Fig 2B). This finding can be explained by the high mobility of domestic goats in relation to human migration and commercial trade [17].

Many studies have investigated the origin of modern domestic livestock, and evidence of high diversity of maternal lineages has been observed in many domesticated animals [42]. The results of the present study demonstrate that five populations of native Omani goats are more closely related to *C*. *aegagrus* than to other wild goats in the phylogenetic tree and thus possess ancient haplotypes. The most ancient rock art in Oman displays wild animals such as ibex, gazelle, ass, auroch and ostrich [7,8,43]. Of significant interest is the presence of ibex-like figures engraved in the rocky walls of many caves and wadis in Oman. These engravings were likely made by hunters that frequented the Jebel Akhdar Mountains in search of prey during the fifth and fourth millennia BC (7,000–6,000 years ago) [8] and might be related to similar figures present elsewhere in the Arabian Peninsula [44,45]. According to the mitochondrial evidence presented herein, the Nubian ibex has not contributed to the gene pool of Omani domestic goats. These speculations might be relevant to analyses of nuclear DNA.

The results of our phylogenetic analysis in combination with 22 goat maternal haplogroup references from GenBank [17] revealed three distinct maternal haplogroups (A, B and G) in domestic goats in Oman (Fig 2A). Haplogroup A is predominant, consistent with the findings of previous studies that indicated that most domesticated goats carry this haplogroup [14,17,18]. Among the other two haplogroups (B and G), B was mostly identified in goats from Pakistan, India and Southeast Asia (Myanmar and Cambodia) and was also present in goat from Sub-Saharan Africa and Europe [15,17,18,46–48], whereas G was observed in individuals from the Near and Middle East, including Saudi Arabia, Iran, and Turkey, as well as in North-East Africa [17,18,41]. The arrival of mtDNA haplogroups to Oman might have occurred through two types of routes: continental routes via the north gateway of the Arabian Peninsula and maritime routes via the Indian Ocean. Clear evidence supporting this hypothesis was obtained within the nine shared haplotypes, with some of the Omani goats showing the same haplotypes as those from neighboring countries, as presented in S7 Table. It can be reasonably concluded that admixture occurred between Omani goats and the populations of nearby countries through commercial trade or during the migratory movements of humans in ancient times [17]. Moreover, Colli et al. [18] and Naderi et al. [23] recently confirmed that all of the mitochondrial DNA haplogroups found in current domestic goats are also present in *C*. *aegagrus* populations in Iran. Although Oman is located on the other side of the Southern Zagros, Central Iranian Plateau and Sistan and Baluchestan Province of Iran, where *C*. *aegagrus* is widely distributed, some early domestic goats in Oman likely acquired mitochondrial genes through the fertilization of *C*. *aegagrus* in these regions.

The comparison of Omani populations with ten other populations from other countries (S6 Table) revealed very limited differentiation. Pairwise comparisons of the genetic distances between the Omani and Arabian goat populations frequently yielded low *F*_*ST*_ values, and the genetic distance typically increased in both value and significance with increasing geographic distance (Fig 1B). Similarly, the *F*_*ST*_ values were correlated with increased or decreased geographic distance, as confirmed by our Mantel test results (Fig 1C). Our analyses also support the suggestion from previous studies that during history, some gene flow occurred between geographically proximate goat populations due to their transport [14,17,30,49].

Thus, information on Arabian history can provide indirect clues regarding the genetic history of goats in the Arabian Peninsula. From a very early period, Arabs had extensive contact with their Near Eastern neighbors, Mesopotamia, the Levant, Egypt and the peoples of the Mediterranean, East African and Indian Subcontinent. These contacts were particularly associated with commerce in frankincense (*Boswellia sacra*), myrrh, copper and animals of southern Arabia. To understand the genetic history of goats in Oman, a brief review of the history of the region, particularly in terms of cultural and population contacts with other peoples, is indispensable. Substantial evidence from Neolithic tools (pottery and ceramic) reveals a historical connection between the Oman Peninsula (Magan and Melukhah Empires) and Mesopotamia (Iraq) since the end of the last glaciation (10,000 years ago) [50]. Burial sites in Oman became elaborate, with the presence of Baluchi/Iranian-style pottery and Harappan beads from the Indus valley, establishing a connection between Oman and present-day Iran/Pakistan [50]. The copper trade flourished in the second millennium BC, with Omani copper mined at Sohar (Magan Empire) being moved through the Dilmun culture and traded in the Mesopotamian city of Ur [50]. Bronze weapons from Nizwa also suggest an Iranian connection [50]. In addition, several Paleolithic sites in Oman have documented techno-typological affinities with industries in the Horn of Africa, the Levant and India, underscoring Arabia’s role as a nexus between continents [51]. Furthermore, there is ample evidence from studies of molecular epidemiology, the Y chromosome and mtDNA to support historical movements of nearby human populations with frequent arrivals in Oman [50–54].

Thus, the apparent lack of significant population structuring among Omani goats and nearby populations can be attributed to ancient contact between Oman and Mesopotamia, southern Arabia and the Indus valley as well as eastern Africa. In addition, goat herds in Oman are reared under two traditional pastoral systems, specifically Badawi (nomadic) and Jabali or Shawawi, i.e., mountain goat herding (semi-nomadic), and the herding and movement characteristics are determined by the availability of food and the climatic conditions. Of course, this type of herding most likely resulted in an increase in apparent admixture with exotic blood. Nonetheless, humans prefer to keep goats during migratory movements because these animals can survive in the most adverse circumstances and supply a full range of useful products [55]. Moreover, people in early times treated goats as currency [21], and this treatment might be responsible for the observed gene flow among different goat populations in different regions.

The estimate of 4,119 years for the relative time since the expansion of the Omani goat shows that although the Zagros mountains, as the center of origin of the domestic goat, are located at the other side of the Arabian Gulf (less than 1,000 km away), extant Omani goats descend from a much more recent expansion event. In addition, we calculated approximate expansion dates of 5,389 years ago for haplogroup A and 6,353 years ago for haplogroup G, which are more recent than the estimates reported by Colli et al. (roughly 12,800 and 9,000 years ago, respectively) [18] and significantly postdate the initiation of domestication (~10,000 years ago). These results are compatible with archaeological evidence for the presence of goats in faunal assemblages at many sites in Oman toward the end of the seventh millennium BC. Furthermore, the timing is nearly concordant with ancient human activities, such as painting and engraving of animals on natural rocks and caves across Oman (approximately 7,000–6,000 years ago).

Archaeological results indicate that animal husbandry appeared in Oman at approximately 7,000–6,500 cal BC and that the Omani goat might have been domesticated during the Neolithic period, between 5,500 and 3,200 cal BC [9,10]. This scenario might be confirmed using ancient domestic goat DNA to clarify and help elucidate the history of goats in the Oman region.

## Conclusions

This study provides the first insights into the genetic diversity and evolutionary background of domestic goats in Oman and provides a basis for further studies on the temporal fluctuations of domestic goat populations. Our results indicate high diversity within the five examined Omani domestic goat populations. Phylogenetic analyses revealed that the five breeds descended from *C*. *aegagrus* and belong to three mtDNA haplogroups (A, B and G). Pairwise *F*_*ST*_ comparisons revealed that populations from geographically proximal sites are more likely to undergo gene exchange. The inclusion of more samples of wild goat species or ancient goats in future studies will provide additional insights into the history and gene flow of goat populations in Oman.

## Supporting information

S1 File Brief description of Omani goat breeds. (DOCX)

S1 Fig Morphological characteristics of five Omani goat populations. (TIF)

S1 Table List of the seven wild goat species included in the phylogenetic analyses. (DOCX)

S2 Table Reference mtDNA sequences of seven goat populations Used in the pairwise *F*_*ST*_ comparisons. (DOCX)

S3 Table Relative geographic distance (km) between pairs of countries. (DOCX)

S4 Table Pairwise differences (*F*_*ST*_) between Omani goat populations based on the 525-bp mtDNA sequence. (DOCX)

S5 Table Omani goat mtDNA variation within and among populations based on an Analysis of Molecular Variation (AMOVA). (DOCX)

S6 Table Comparisons of population pairwise *F*_*ST*_ values between Omani goats and nine other goat populations. (DOCX)

S7 Table Distribution of haplotype sharing with neighboring countries. (DOCX)
