# Supplementary material for: Genetic origin of goat populations in Oman revealed by mitochondrial DNA analysis
Source: PLoS One. 2017 Dec 27;12(12):e0190235. doi: 10.1371/journal.pone.0190235 (PMC5744987; doi:10.1371/journal.pone.0190235)
Supplement: S1 File — (DOCX) [file pone.0190235.s001.docx]

**S1 File.** **Brief descriptions of Omani goat breeds**

The Jabal Akhdar breed (JKH) is mostly found at high elevation (3,048 m a.s.l.) in the interior region of Al Jabal Al Akhdar (Green Mountain). It has back-twisted horns in both sexes, small ears and long soft hair dominated by a white to gold color. It has fast growth rates; a heavier body, carcass weight and carcass yield; and good meat quality [1-3], making it more attractive for meat production and breeding than are other goat breeds. The Batinah goat breed (BTN) is widely distributed in the north coastal plain of the Al Batinah region. This breed can survive and resist the extreme humidity and heat in the summer months on the north coastal plains [4]. It is often covered with thick long brown or reddish brown hair. The third breed, Dhofari (DHR), is mostly found in the hills of Dhofar in southern Oman, where the highest densities of cattle and camel herds are found, and it is well adapted for grazing in wet conditions. Dhofari goats come in a variety of colors, but the pure white color is the most common. It has a higher milk yield than the Jabal Akhdar and Batinah breeds [5]. It is used for meat in Dhofar province and is smaller than other Omani breeds, although it is also more efficient at feed conversion, with a lower cost for meat production than imported goats [6]. The last breed is an Ash Sharqiyah goat (SHR), mostly found in three different geographic areas of the Ash Sharqiyah district: the sands of the eastern region, the areas near the mountains and the coastal areas of southern Ash Sharqiyah. The breed is very well adapted for long migration, poor nutrition and drought, and it is resistant to parasitic diseases. Thus, this breed is important for meat production in the arid areas of Oman because of its high survival and reproductive rates on natural grazing rangelands. The Ash Sharqiyah breed has a short, rough, hairy coat. The most common color of this breed is black with two white or brown stripes on the face and white or brown on the abdomen, resembling Alpine breeds (S1 Fig). However, black or white and brown/fawn animals are also found. Finally, the Musandam strain (MSN) is predominantly black or white with black markings. Both sexes have wattles and back-twisted horns. It is phenotypically similar to the Ash Sharqiyah breed and is found in northern Oman, on the Musandam peninsula, which juts into the Strait of Hormuz, the narrow entry into the Arabian/Persian Gulf from the Arabian Peninsula.

**References**

1. Kadim I, Mahgoub O, Al-Ajmi D, Al-Maqbaly R, Al-Saqri N, Ritchie A. An evaluation of the growth, carcass and meat quality characteristics of Omani goat breeds. Meat Science. 2004;66(1):203-10.

2. Mahgoub O, Kadim I, Al-Saqry N, Al-Busaidi R. Potential of Omani Jebel Akhdar goat for meat production under feedlot conditions. Small Ruminant Research. 2005;56(1):223-30.

3. Al-Nakib F, Al-Shukaily E, Al-Hanai S, Al-Nabhani S. Comparative performance of Omani goats and sheep. The Journal of Agricultural Science. 1996;127(1):117-21.

4. Srikandakumar A, Johnson E, Mahgoub O. Effect of heat stress on respiratory rate, rectal temperature and blood chemistry in Omani and Australian Merino sheep. Small Ruminant Research. 2003;49(2):193-8.

5. Chesworth J, Horton G. Lactation in indigenous Omani goats. International Journal of Animal Sciences. 1996;11:7-12.

6. El Hag M, El Shargi K. Feedlot performance and carcass characteristics of local (Dhofari) and exotic (Cashmere) goats fed on a high-fiber by-products diet supplemented with fish sardine. ASIAN AUSTRALASIAN JOURNAL OF ANIMAL SCIENCES. 1996;9:389-96.
