# Supplementary figures and images for: Genetic origin of goat populations in Oman revealed by mitochondrial DNA analysis
Source: PLoS One. 2017 Dec 27;12(12):e0190235. doi: 10.1371/journal.pone.0190235 (PMC5744987; doi:10.1371/journal.pone.0190235)

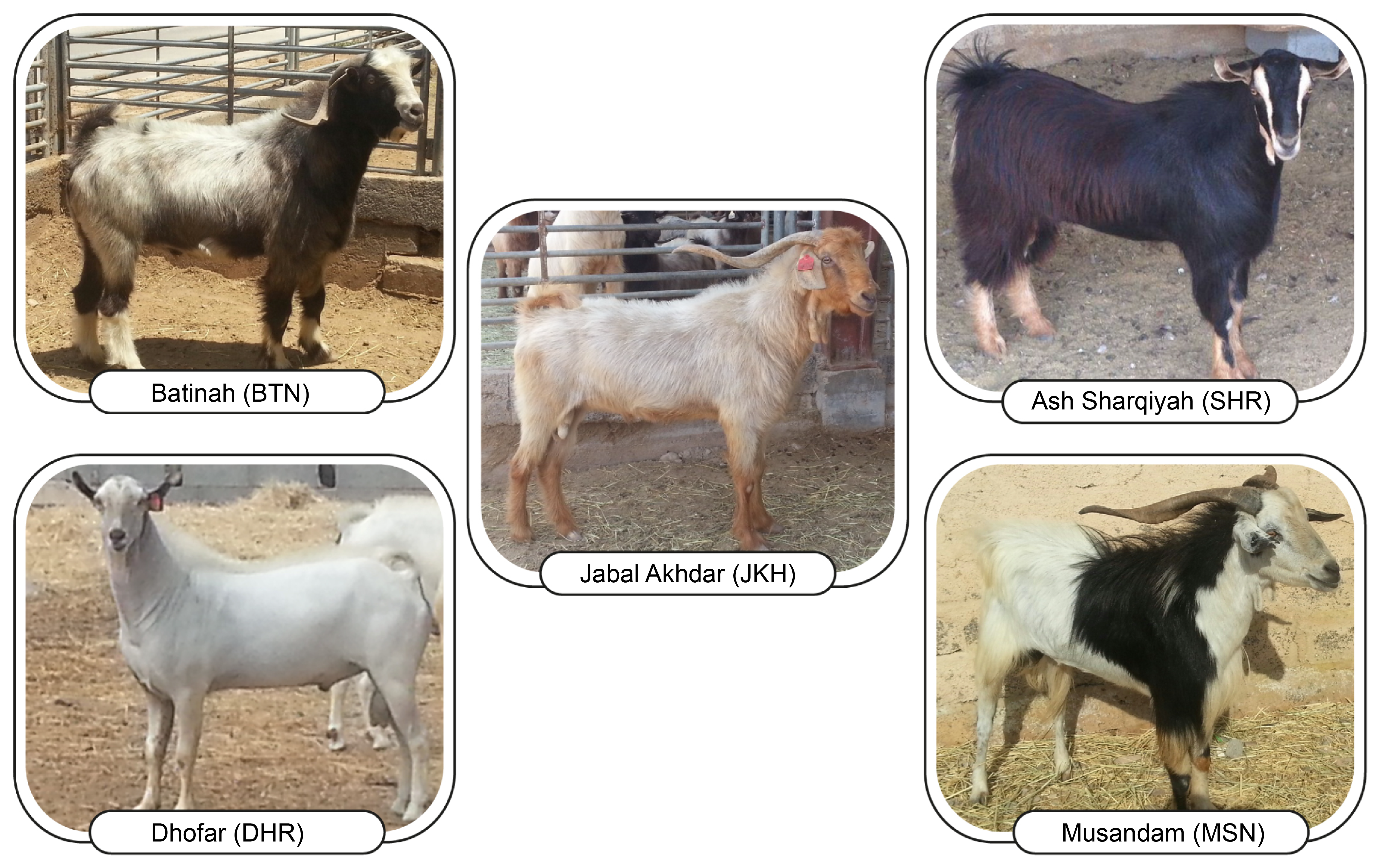

Supplement: S1 Fig — (TIF) [file pone.0190235.s002.tif]
