# Supplementary material for: Genetic origin of goat populations in Oman revealed by mitochondrial DNA analysis
Source: PLoS One. 2017 Dec 27;12(12):e0190235. doi: 10.1371/journal.pone.0190235 (PMC5744987; doi:10.1371/journal.pone.0190235)
Supplement: S1 Table — (DOCX) [file pone.0190235.s003.docx]

**S1 Table. List of the seven wild goat species included in the phylogenetic analyses**

| Species | Common name | Code in Fig 2C | Accession numbers |
| --- | --- | --- | --- |
| *Capra aegagrus* | Bezoar | CAG001 | AJ317864 [1] |
|  |  | CAG002 | AJ317865 [1] |
|  |  | CAG003 | AJ317866 [1] |
|  |  | CAG004 | EF989498 [2] |
|  |  | CAG005 | EF989645 [2] |
|  | Sindh ibex | CAG007 | AB110591[3] |
|  |  | CAG007 | AB110590[3] |
| *Capra falconeri* | Markhor | CFA001 | AJ317873 [1] |
|  |  | CFA002 | AJ317872 [1] |
|  |  | CFA003 | AB044305[4] |
|  |  | CFA004 | AB044306[4] |
| *Capra cylindricornis* | East Caucasian tur | CCY001 | AJ317868 [1] |
|  | East Caucasian tur | CCY002 | AJ317869 [1] |
|  | East Caucasian tur | CCY003 | AJ317870 [1] |
| *Capra [ibex] nubiana* | Nubian ibex | CNU001 | AJ317871 [1] |
| *Arabitragus jayakari* | Arabian tahr | ARJ001 | FJ207523 [5] |
| *Capra [ibex] caucasica* | West Caucasian tur | CCA001 | AJ317875 [1] |
| *Capra [ibex] sibirica* | Siberian ibex | CSI001 | AJ317874 [1] |

**References**

1. Luikart G, Gielly L, Excoffier L, Vigne J-D, Bouvet J, Taberlet P. Multiple maternal origins and weak phylogeographic structure in domestic goats. Proc Natl Acad Sci U S A. 2001;98(10):5927-32.

2. Naderi S, Rezaei H-R, Taberlet P, Zundel S, Rafat S-A, Naghash H-R, et al. Large-Scale Mitochondrial DNA Analysis of the Domestic Goat Reveals Six Haplogroups with High Diversity. PLoS ONE. 2007;2(10):e1012.

3. Sultana S, Mannen H, Tsuji S. Mitochondrial DNA diversity of Pakistani goats. Anim Genet. 2003;34(6):417-21.

4. Mannen H, Nagata Y, Tsuji S. Mitochondrial DNA reveal that domestic goat (*Capra hircus*) are genetically affected by two subspecies of bezoar (*Capra aegagurus*). Biochem Genet. 2001;39(5-6):145-54.

5. Hassanin A, Ropiquet A, Couloux A, Cruaud C. Evolution of the Mitochondrial Genome in Mammals Living at High Altitude: New Insights from a Study of the Tribe Caprini (Bovidae, Antilopinae). J Mol Evol. 2009;68(4):293-310.
