# Supplementary material for: Genetic origin of goat populations in Oman revealed by mitochondrial DNA analysis
Source: PLoS One. 2017 Dec 27;12(12):e0190235. doi: 10.1371/journal.pone.0190235 (PMC5744987; doi:10.1371/journal.pone.0190235)
Supplement: S2 Table — (DOCX) [file pone.0190235.s004.docx]

**S2 Table. Reference mtDNA sequences of seven goat populations used in pairwise F_ST_ comparisons**

| Population | Code | No. of sequences | Accession numbers and citation |
| --- | --- | --- | --- |
| Egypt | EGY | 29 | AJ317780 - AJ317783; AJ317795 - AJ317801 [1]; EF617711 - EF617728 [2] |
| Iraq | IRQ | 7 | AJ317762 - AJ317768 [1] |
| Saudi Arabia | SAU | 44 | AJ317752 - AJ317759 [1]; EF618309 - EF618339; EF618341 - EF618345 [2] |
| Iran | IRN | 219 | EF617863 - EF618067; EF618070 - EF618084 [2] |
| Pakistan | PAK | 88 | AB162196 - AB162200; AB162202 - AB162205; AB162208 - AB162212; AB162214 - AB162217 [3]; AJ317826; AJ317533; AJ317554 - AJ317555; AJ317557 - AJ317559; AJ317563 - AJ317565; AJ317845 - AJ317850; AJ317858 - AJ317863 [1]; EF618253 - EF618263 [2]; AB110561 - AB110589 [4] |
| India | IND | 462 | KC817830 - KC817862; KC817907 - KC817923; KC817976 - KC817991; KC818011 - KC818026 [5]; AJ317540 - AJ317544; AJ317560 - AJ317562; AJ317571 - AJ317572; AJ317827; AJ317830; AJ317856 - AJ317857 [1]; AY155674 - AY156039 [6]; EF617856 - EF617862 [2] |
| Turkey | TUR | 349 | KC574086 - KC574380 [7]; AJ317736 - AJ317751; AJ317842 - AJ317843 [1]; EF618501 -EF618534; EF618536 - EF618539 [2] |

**References**

1. Luikart G, Gielly L, Excoffier L, Vigne J-D, Bouvet J, Taberlet P. Multiple maternal origins and weak phylogeographic structure in domestic goats. Proc Natl Acad Sci U S A. 2001;98(10):5927-32.

2. Naderi S, Rezaei H-R, Taberlet P, Zundel S, Rafat S-A, Naghash H-R, et al. Large-scale mitochondrial DNA analysis of the domestic goat reveals six haplogroups with high diversity. PLoS ONE. 2007;2(10):e1012.

3. Sultana S, Mannen H. Polymorphism and evolutionary profile of mitochondrial DNA control region inferred from the sequences of Pakistani goats. Anim Sci J. 2004;75(4):303-9.

4. Sultana S, Mannen H, Tsuji S. Mitochondrial DNA diversity of Pakistani goats. Anim Genet. 2003;34(6):417-21.

5. Rana S, Singh S, Dureja V, Joshi J, Banerjee P, Sharma U, et al. Mitochondrial D-loop analysis reveals single maternal lineage for coastal region goats of India. DHR International Journal of Biomedical and Life Sciences. 2013;4(1):235-44.

6. Joshi MB, Rout PK, Mandal AK, Tyler-Smith C, Singh L, Thangaraj K. Phylogeography and origin of Indian domestic goats. Mol Biol Evol. 2004;21(3):454-62.

7. Akis I, Oztabak K, Mengi A, Un C. Mitochondrial DNA diversity of Anatolian indigenous domestic goats. J Anim Breed Genet. 2014;131(6):487-95.
