# Supplementary material for: Genetic origin of goat populations in Oman revealed by mitochondrial DNA analysis
Source: PLoS One. 2017 Dec 27;12(12):e0190235. doi: 10.1371/journal.pone.0190235 (PMC5744987; doi:10.1371/journal.pone.0190235)
Supplement: S3 Table — (DOCX) [file pone.0190235.s005.docx]

**S3 Table. Relative geographic distance (km) between pairs of countries**

| Population | OMN | IRQ | YEM | SAU | SOM | EGY | IRN | TUR | PAK | IND |
| --- | --- | --- | --- | --- | --- | --- | --- | --- | --- | --- |
| OMN | 0 |  |  |  |  |  |  |  |  |  |
| IRQ | 1776 | 0 |  |  |  |  |  |  |  |  |
| YEM | 1025 | 2027 | 0 |  |  |  |  |  |  |  |
| SAU | 1144 | 1048 | 995 | 0 |  |  |  |  |  |  |
| SOM | 2102 | 3136 | 1185 | 2089 | 0 |  |  |  |  |  |
| EGY | 2614 | 1430 | 2221 | 1472 | 2914 | 0 |  |  |  |  |
| IRN | 1235 | 940 | 1950 | 1271 | 3135 | 2296 | 0 |  |  |  |
| TUR | 2767 | 991 | 2911 | 1918 | 3923 | 1414 | 1815 | 0 |  |  |
| PAK | 1665 | 2443 | 2689 | 2504 | 3711 | 3770 | 1504 | 3244 | 0 |  |
| IND | 2394 | 3751 | 3266 | 3502 | 3936 | 4929 | 2831 | 4636 | 1454 | 0 |

Country names are abbreviated as Oman (OMN), Iraq (IRQ), Yemen (YEM), Saudi Arabia (SAU), Somalia (SOM), Egypt (EGY), Iran (IRN), Turkey (TUR), Pakistan (PAK) and India (IND).
