# Supplementary material for: Genetic origin of goat populations in Oman revealed by mitochondrial DNA analysis
Source: PLoS One. 2017 Dec 27;12(12):e0190235. doi: 10.1371/journal.pone.0190235 (PMC5744987; doi:10.1371/journal.pone.0190235)
Supplement: S4 Table — (DOCX) [file pone.0190235.s006.docx]

**S4 Table. Pairwise differences (*F_ST_*) between Omani goat populations based on the 525-bp mtDNA sequence**

| Population | JKH | BTN | DHR | SHR | MSN |
| --- | --- | --- | --- | --- | --- |
| JKH | 0 |  |  |  |  |
| BTN | 0.063 | 0 |  |  |  |
| DHR | 0.043 | 0.005 | 0 |  |  |
| SHR | -0.027 | 0.069 | 0.064 | 0 |  |
| MSN | 0.097* | 0.155* | 0.163* | 0.038 | 0 |

The negative F_ST_ value in the comparison of Jabal Akhdar (JKH) and Ash Sharqiyah (SHR) should be interpreted as zero. * Significant values (P-value ˂ 0.05). Population names are abbreviated as Jabal Akhdar (JKH), Batinah (BTN), Dhofar (DHR), Ash Sharqiyah (SHR) and Musandam (MSN).
