# Supplementary material for: Genetic origin of goat populations in Oman revealed by mitochondrial DNA analysis
Source: PLoS One. 2017 Dec 27;12(12):e0190235. doi: 10.1371/journal.pone.0190235 (PMC5744987; doi:10.1371/journal.pone.0190235)
Supplement: S5 Table — (DOCX) [file pone.0190235.s007.docx]

**S5 Table. Omani goat mtDNA variation within and among populations based on an analysis of molecular variation (AMOVA)**

| Source of variation | Degrees of freedom | Sum of squares | Variance components | Percentage of variation | *F*_ST_ | *P*-value |
| --- | --- | --- | --- | --- | --- | --- |
| Among populations | 4 | 53.006 | 0.453 | 6.02* | 0.060 | 0.006 |
| Within populations | 64 | 453.023 | 7.079 | 93.98* |  |  |
| Total | 68 | 506.029 | 7.532 | 100 |  |  |

* Significant values (P-value ˂ 0.05); *P*-values were obtained based on 1000 permutations.
