# Supplementary material for: Genetic origin of goat populations in Oman revealed by mitochondrial DNA analysis
Source: PLoS One. 2017 Dec 27;12(12):e0190235. doi: 10.1371/journal.pone.0190235 (PMC5744987; doi:10.1371/journal.pone.0190235)
Supplement: S6 Table — (DOCX) [file pone.0190235.s008.docx]

**S6 Table. Comparisons of population pairwise *F_ST_* values between Omani goats and nine other goat populations**

| Population | OMN | IRQ | YEM | SAU | SOM | EGY | IRN | TUR | PAK | IND |
| --- | --- | --- | --- | --- | --- | --- | --- | --- | --- | --- |
| OMN | 0 |  |  |  |  |  |  |  |  |  |
| IRQ | 0.017 | 0 |  |  |  |  |  |  |  |  |
| YEM | 0.019 | - 0.024 | 0 |  |  |  |  |  |  |  |
| SAU | 0.015 | 0.014 | - 0.004 | 0 |  |  |  |  |  |  |
| SOM | - 0.031 | - 0.009 | 0.002 | - 0.023 | 0 |  |  |  |  |  |
| EGY | 0.024* | 0.007 | 0.007 | 0.009 | 0.013 | 0 |  |  |  |  |
| IRN | 0.045*** | - 0.012 | 0.035* | 0.040*** | 0.027 | 0.023** | 0 |  |  |  |
| TUR | 0.058*** | - 0.005 | 0.043* | 0.050*** | 0.042* | 0.037*** | 0.011*** | 0 |  |  |
| PAK | 0.063*** | 0.030 | 0.066* | 0.093*** | 0.055 | 0.085*** | 0.059*** | 0.078*** | 0 |  |
| IND | 0.134*** | 0.059* | 0.114*** | 0.139*** | 0.150** | 0.107*** | 0.060*** | 0.085*** | 0.045*** | 0 |

Pairwise difference was used as the distance method. The published goat mtDNA sequences used in this study for goat population pairwise *F_ST_* values are given in S2 Table. Statistical significance is indicated as follows: * P-value ˂ 0.05; ** P-value ˂ 0.01; *** P-value ˂ 0.001. Goat population names are abbreviated as Oman (OMN), Iraq (IRQ), Yemen (YEM), Saudi Arabia (SAU), Somalia (SOM), Egypt (EGY), Iran (IRN), Turkey (TUR), Pakistan (PAK) and India (IND).
