# Supplementary material for: Genetic origin of goat populations in Oman revealed by mitochondrial DNA analysis
Source: PLoS One. 2017 Dec 27;12(12):e0190235. doi: 10.1371/journal.pone.0190235 (PMC5744987; doi:10.1371/journal.pone.0190235)
Supplement: S7 Table — (DOCX) [file pone.0190235.s009.docx]

**S7 Table. Distribution of haplotype sharing with neighboring countries**

| Haplotypes | Omani Breeds (n)^a^ | Countries (n) | Haplogroups |
| --- | --- | --- | --- |
| H3 | Batinah (1) | Iran (1) | A |
| H7 | Batinah (1) | Egypt (1) | A |
| H9 | Dhofar (3) | Turkey (3) | G |
| H16 | Dhofar (1) | Pakistan (1) | A |
| H19 | Jabal Akhdar (2) | Iran (2); Turkey (1) | A |
| H25 | Jabal Akhdar (1); Musandam (1) | Iran (1); Turkey (1) | A |
| H32 | Jabal Akhdar (3); Ash Sharqiyah (1) | no | B |
| H36 | Musandam (1) | India (1) | A |
| H37 | Musandam (2) | Iran (1); India (7); Pakistan (1) | A |
| H44 | Ash Sharqiyah (2) | Iran (2) | A |

^a^ Number of individuals
